# Molecular mimicry among human proteinase 3 and bacterial antigens: implications for development of c-ANCA associated vasculitis

**DOI:** 10.1093/oxfimm/iqac009

**Published:** 2022-12-02

**Authors:** Y Chavez, J Garces, R Díaz, M Escobar, A Sanchez, E Buendía, M Múnera

**Affiliations:** Medical Research Group (GINUMED) Universitary Corporation Rafael Nuñez, Centro Calle de la Soledad No. 5–70, Cartagena 130002, Colombia; Medical Research Group (GINUMED) Universitary Corporation Rafael Nuñez, Centro Calle de la Soledad No. 5–70, Cartagena 130002, Colombia; Medical Research Group (GINUMED) Universitary Corporation Rafael Nuñez, Centro Calle de la Soledad No. 5–70, Cartagena 130002, Colombia; Catholic University of Manizales, Manizales, Av. Santander #No. 60, Manizales, Caldas 111321, Colombia; Medical Research Group (GINUMED) Universitary Corporation Rafael Nuñez, Centro Calle de la Soledad No. 5–70, Cartagena 130002, Colombia; Group of Clinical and Experimental Allergy (GACE), IPS Universitaria, University of Antioquia, Cl 77 y 78 al norte, Antioquia, Medellín 050010, Colombia; Faculty of Medicine, University of Cartagena, Cra. 50 #24120, Zaragocilla, Cartagena de Indias, Provincia de Cartagena, Bolívar, Cartagena 130014, Colombia; Clinical and Biomedical Research Group, Faculty of Medicine, University of Cartagena, Cra. 50 #24120, Zaragocilla, Cartagena de Indias, Provincia de Cartagena, Bolívar, Cartagena 130014, Colombia; Serena del Mar Hospital Center, VIA AL MAR KM 8, Cartagena de Indias, Provincia de Cartagena, Bolívar, Cartagena 130008, Colombia; Medical Research Group (GINUMED) Universitary Corporation Rafael Nuñez, Centro Calle de la Soledad No. 5–70, Cartagena 130002, Colombia

**Keywords:** autoantigen, autoimmunity, cross-reactivity, microbial, PR3, molecular mimicry

## Abstract

Wegener’s granulomatosis is an autoimmune disease where autoantibodies target human autoantigen PR3, a serine protease locates on the neutrophil membrane. This disease affects blood small vessels and could be deadly. The origin of these autoantibodies is unknown, but infections have been implicated with autoimmune disease. In this study, we explored potential molecular mimicry between human PR3 and homologous pathogens through *in silico* analysis. Thirteen serine proteases from human pathogens (*Klebsiella pneumoniae*, *Acinetobacter baumannii*, *Salmonella* sp., *Streptococcus suis*, *Vibrio parahaemolyticus*, *Bacteroides fragilis*, *Enterobacter ludwigii*, *Vibrio alginolyticus*, *Staphylococcus haemolyticus*, *Enterobacter cloacae*, *Escherichia coli* and *Pseudomonas aeruginosa*) shared structural homology and amino acid sequence identity with human PR3. Epitope prediction found an only conserved epitope IVGG, located between residues 59–74. However, multiple alignments showed conserved regions that could be involved in cross-reactivity between human and pathogens serine proteases (90–98, 101–108, 162–169, 267 and 262 residues positions). In conclusion, this is the first report providing *in silico* evidence about the existence of molecular mimicry between human and pathogens serine proteases, that could explain the origins of autoantibodies found in patients suffering from Wegener’s granulomatosis.

## Graphical Abstract

**Figure:**
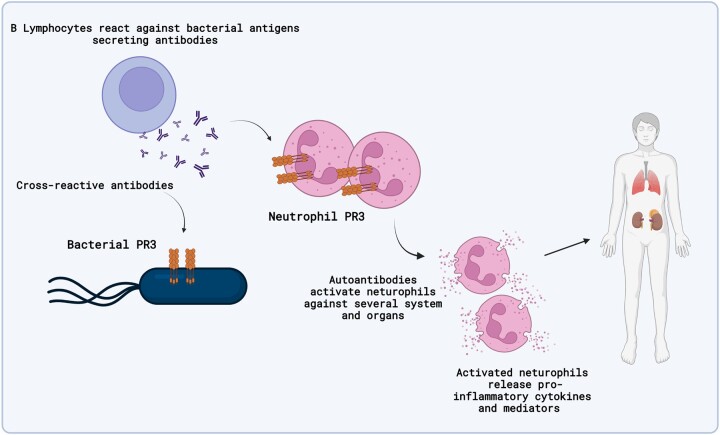

## Background

Human proteinase 3 (PR3) is an important autoantigen implicated in the genesis of c-Antineutrophil cytoplasmic antibodies (ANCA) associated vasculitis. Physiologically PR3 is an endogenous protease expressed on the neutrophil membrane that degrades various extracellular matrix proteins and participates in transendothelial migration; however, some individuals produce autoantibodies that target it and after immunotolerance losing, those antibodies can trigger strong inflammatory response against small blood vessels, giving origin to small vessel vasculitis diseases. There is a link between infections and autoimmune diseases [1, 2] and molecular mimicry between human and bacterial antigens has been implicated as a potential environmental trigger of autoantibodies [3] and autoimmunity [4]. Indeed, prolonged infections have been associated with the development of antibodies specific to proteinase 3 and vasculitis development [5, 6], but the bacterial components triggering the production of autoantibodies have not been described for this disease.

The study of autoimmunity can use several approaches, including epidemiological, proteomic, serologic and *in silico* tools, being the last one a preliminary step to determine if there is molecular mimicry between two antigens belonging to two not related biological sources [7]. Previously, Pendergraft *et al.* [8] searched for microbial or fungal mimics of PR3 resulted and found no hits, but with the advent of constantly curated peptide databases [9], bacterial proteins reported sequences have increased without precedents making possible an updated *in silico* approach. Here, we have explored molecular mimicry between PR3 and bacterial antigens using *in silico* approaches, also, structural, and antigenic prediction were performed. We found that PR3 is a well-conservated protein in bacterial microorganisms of clinical interest, and conservation support existence of molecular mimicry that could explain how infection impact in the development of autoimmunity in c-ANCA associated vasculitis.

## Materials and methods

### Antigens retrieve

Amino acid sequence of human PR3 (hPR3) was retrieved from Uniprot database with accession number: P24158 [9], which was used as input in PSI-BLASTp to search bacterial homologous by using identifier Bacteria (taxid: 2). General parameters were set up by default. Amino acid sequences from bacteria with clinical relevance for human were used for further analysis.

### Multiple alignment analysis

The objective of multiple alignment is to compare the amino acid sequences of different proteins used in this study, and thus, to understand the level of identity that exists between them, as well as to identify in which regions of these sequences a certain level of conservation occurs, which can explain molecular mimicry. BIVU PRALINE tool was used to perform multiple alignment of amino acid sequences among hPR3 and its bacterial homologous [10]. BLOSUM62 was used as Exchange weights matrix, other parameters were set up as default.

### Modelling based on homology

Some proteins do not have their 3D structure resolved by experimental methods; homology-based modelling allows us in this study to have all the structures of the homologs to PR3. hPR3 3D structure was retrieved from the Protein Data Base with PDB identifier: 1FUJ. Tridimensional structures for serine protease from bacteria were determined by modelling based on homology using Swiss Model server [11]. UCSF chimera allowed to perform root median square deviation (RMSD) analysis and Pymol software was used to visualize models [11, 12].

### Evolutionary analysis

Consurf tool [13] was used to calculate evolutionary conservation of PR3, and identify regions conserved that could help to explain molecular mimicry among hPR3 and homologous in bacterial. Algorithm HMMER and one iteration with an *E*-value cutoff of 0.0001 were used as default parameters.

### Epitope prediction

Ellipro and Bepipred servers were used to predict epitopes on hPR3 [14, 15]. Epitopes with amino acid sequences conserved among human and bacteria were reported.

## Results

### Antigen selection

**Figure 1:**
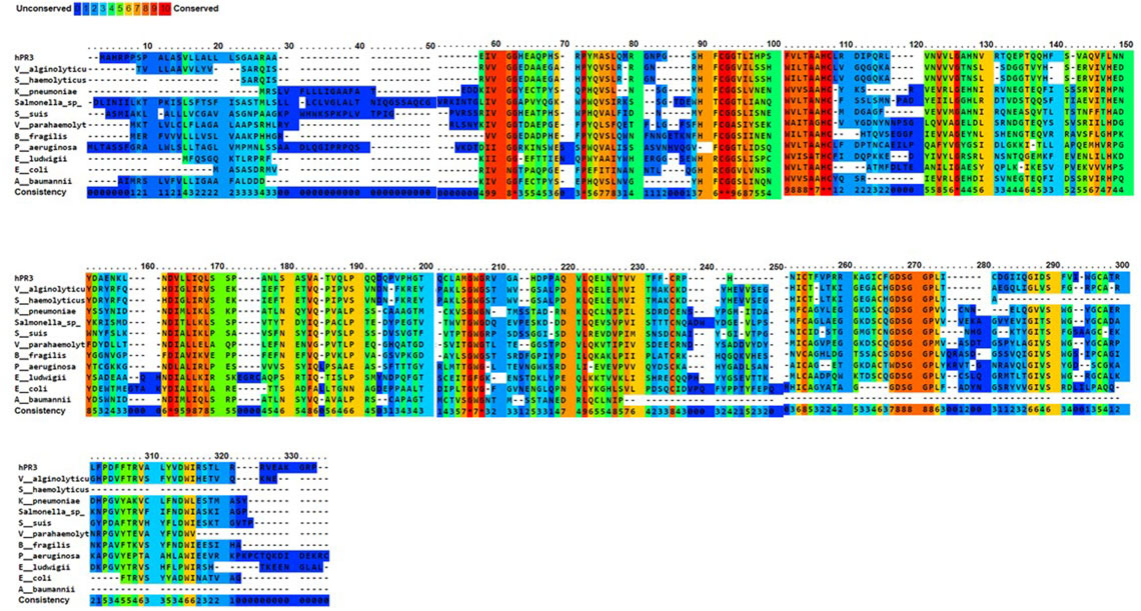
Multiple alignment among serine proteases from human and pathogens. An identity level of 21% was found among all amino acid sequences compared with hPR3.

In total, we found identity between hPR3 and serine protease from 11 pathogens with potential clinical relevance for human (Table 1). In binary alignment between hPR3 and individual serine protease from pathogens, a minimum identity of 24% was found (Table 1). Multiple alignment showed an overall identity among all serine proteases used in this study of 34% (Fig. 1).

**Table 1:** Identity among aminoacid sequences from PR3 and homologous from bacterial are shown

Bacterial species	Uniprot	Identity (%)
*Klebsiella pneumoniae*	WP_172717911.1	36.4
*Acinetobacter baumannii*	WP_139162030.1	40.3
*Salmonella* sp.	WP_187787866.1	31.3
*Streptococcus suis*	WP_203200685.1	37
*Vibrio parahaemolyticus*	WP_180804940.1	29.8
*Bacteroides fragilis*	WP_151874154.1	32.6
*Enterobacter ludwigii*	WP_020883487.1	30.4
*Vibrio alginolyticus*	WP_064377522.1	34.9
*Staphylococcus haemolyticus*	WP_230197895.1	31
*Escherichia coli*	MCI3751435.1	29.2
*Pseudomonas aeruginosa*	WP_033956105.1	24

### Modelling and structural analysis

**Figure 2:**
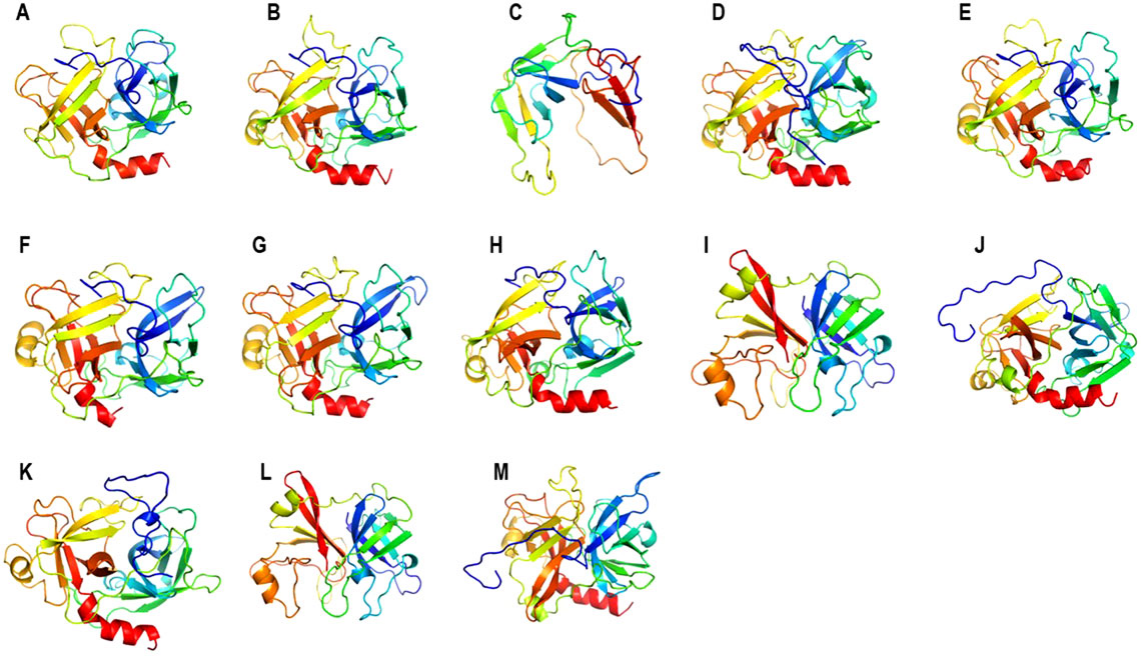
Tridimensional models of bacterial serine proteases. All models exhibited a typical fold of serine protease family. (**A**) *Homo sapiens*, (**B**) *Klebsiella pneumoniae*, (**C**) *Acinetobacter baumannii* (**D**) *Salmonella* spp. (**E**) *Streptococcus suis*, (**F**) *Vibrio parahaemolyticus*, (**G**) *Bacteroides fragilis*, (**H**) *Enterobacter ludwigii*, (**I**) *Vibrio alginolyticus*, (**J**) *Staphylococcus haemolyticus*, (**K**) *Enterobacter cloacae*, (**L**) *Escherichia coli* and (**M**) *Pseudomonas aeruginosa.*

**Figure 3:**
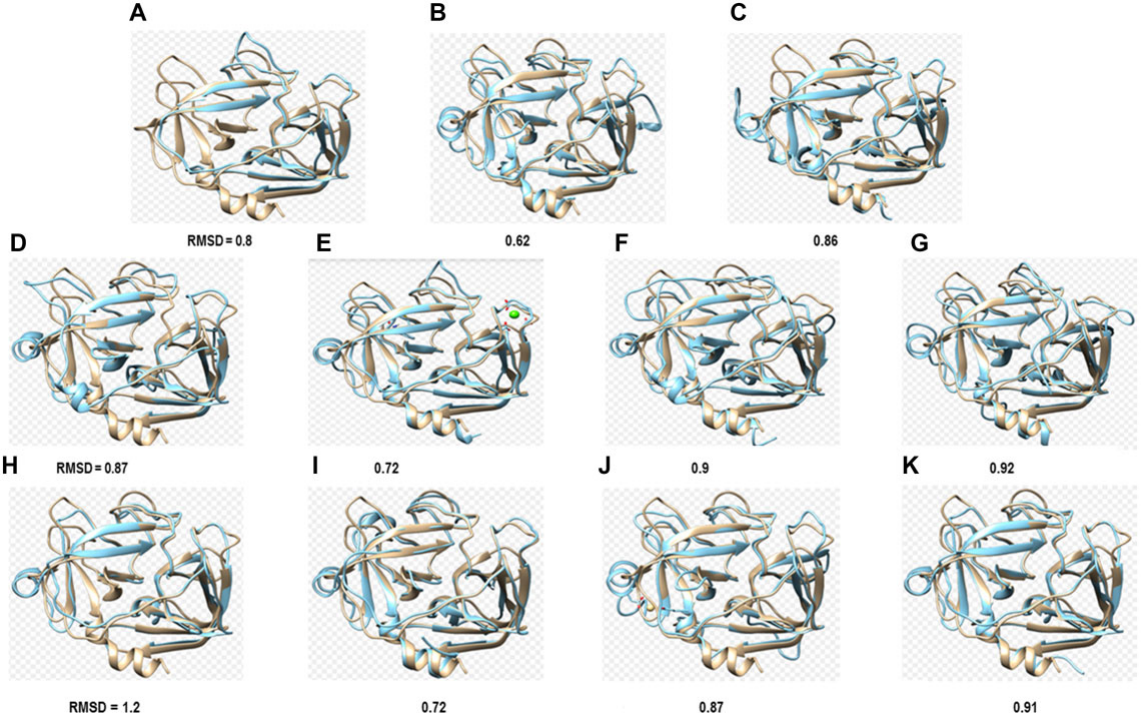
RMSD among hPR3 (brown) and serine proteases from bacterial pathogens (blue). (**A**) PR3 versus antigen of *A. baumannii*, (**B**) *B. fragilis*, (**C**) *E. cloacae*, (**D**) *E. ludwigii*, (**E**) *E. coli*, (**F**) *K. pneumoniae*, (**G**) *P. aeruginosa*, (**H**) *Salmonella* sp., (**I**) *S. haemolyticus*, (**J**) *S. suis*, (**K**) *V. parahaemolyticus* and (L) *V. alginolyticus.*

**Figure 4:**
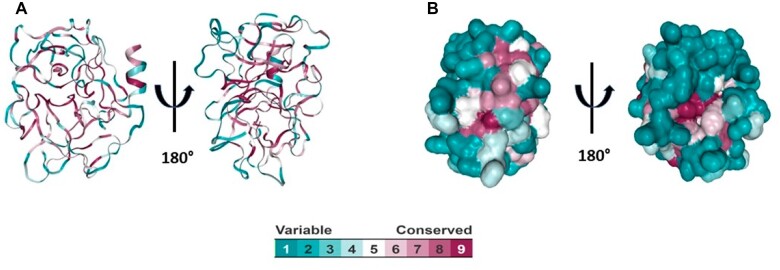
Evolutionary analysis of serine proteases. (**A**, **B**) Cartoon and surface models showing the conserved region among human and bacterial serine proteases.

All serine proteases (except serine proteases from *Salmonella* spp. and *Vibrio alginolyticus*) modelled exhibited a typical fold of this family of proteins with 12-beta strands and 3-alpha helixes (Fig. 2). RMSD analysis showed that hPR3 shares structural homology with serine proteases from bacterial microorganisms, with RMSD values of 0.7. Highest fit was found in beta strands from all serine proteases (Table 1 and Fig. 3). Consurf analysis confirmed that hPR3 belongs to a high conserved family of proteins, highest conservation was found located in beta strands (Fig. 4).

### Epitope prediction

**Figure 5:**
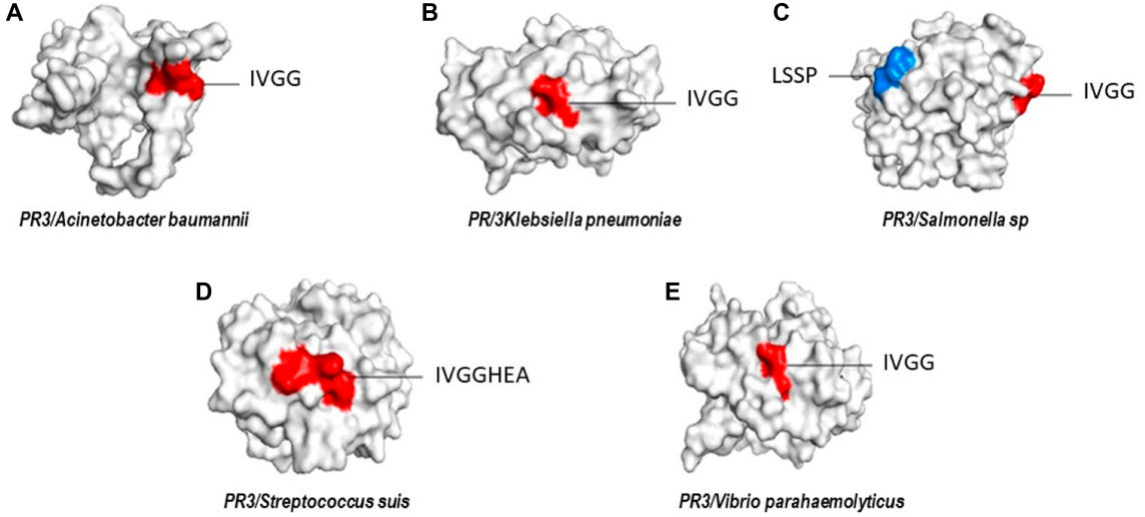
Surface models showed conserved epitopes among hPR3 and homologous in pathogens. (**A**) PR3 from *A. baumannii*, (**B**) *K. pneumoniae*, (**C**) *Salmonella* sp., (**D**) *S. suis* and (**E**) *V. parahaemolyticus.*

Epitope prediction was performed by using hPR3 as input, based on this, one cross-reactive epitope was predicted, with amino acid sequence: IVGG, located between residues 59–74 (Fig. 5).

## Discussion

Molecular mimicry and cross-reactivity between human and bacterial antigens could explain why some genetically predisposed individuals could develop autoimmunity after bacterial infections [3, 4]. Previously, Pendergraft *et al.* [8] reported no microbial or fungal mimics for hPR3 antigen after searching homologs in databases of that time; however, peptide sequence database incompleteness could be missing some relevant bacterial peptides homologies. Here, we found for the first time that hPR3, an autoantigen involved in c-ANCA associated vasculitis share sequence, structural homology and identity with various bacterial serine proteases.

Epitope prediction tools found a potential cross-reactive epitope. However, multiple sequence alignment showed several conserved amino acid sequences: for example, regions located between residues 90–98, 101–108, 162–169, 267 and 262 (Fig. 1) that could also be of relevance. Since this study is a preliminary approach to solve the question of environmental triggers of autoimmunity in the c-ANCA vasculitis field, experimental evidence is awaited as was shown for other autoimmune diseases such as lupus [4]. In conclusion, we report *in silico* evidence that suggest cross-reactivity between hPR3 and various bacterial serine proteases.

Here, we hypothesize that antibodies against serine proteases from pathogens could cross-react with hPR3, this could trigger WG activating neutrophils, which are critical cellular players in this disease, generating a strong inflammatory response that affects blood small vessels [16].

## Data Availability

There is no data available. *Conflict of interest*: All authors declare not have any conflict of interest.
