# Correction to: Molecular mimicry among human proteinase 3 and bacterial antigens: implications for development of c-ANCA associated vasculitis

**DOI:** 10.1093/oxfimm/iqac011

**Published:** 2022-12-27

This is a correction to: Y Chavez, J Garces, M Escobar, A Sanchez, E Buendía, M Múnera, Molecular mimicry among human proteinase 3 and bacterial antigens: implications for development of c-ANCA associated vasculitis, *Oxford Open Immunology*, Volume 3, Issue 1, 2022, iqac009, https://doi.org/10.1093/oxfimm/iqac009

In the originally published version of this manuscript, the third author, Rafael Díaz, was accidently omitted from the final version. This error has been corrected online.

